# Individual differences in affect: explaining work environment perceptions and later wellbeing

**DOI:** 10.1038/s41598-026-55924-9

**Published:** 2026-06-11

**Authors:** Lucas H. Craven, Petri J. Kajonius

**Affiliations:** 1 Department of Psychology, Lund University, Lund, Sweden; 2 Danish National Research Centre for the Working Environment, Copenhagen, Denmark

**Keywords:** Work environment, Individual differences, Well-being, Negative affect, Positive affect, Environmental social sciences, Psychology, Psychology

## Abstract

Individual differences in employees’ affective dispositions influence how they perceive the work environment and report their well-being. A recurring challenge in this literature is that self-reported measures of working conditions and well-being cannot easily distinguish objective environmental features from the perceptual lens through which employees report them. Using a large, stratified sample of Danish employees (T1 *N* = 3970; T2 *N* = 2375) and the Danish Psychosocial Questionnaire, we quantified the extent to which positive and negative affect account for associations between work environment perceptions and employee well-being. Partial correlations, both cross-sectional and longitudinal over six months, showed that two brief affect measures accounted for an average of 73–75% of the observed associations. These findings suggest that a substantial portion of commonly reported work environment-well-being associations reflects stable individual differences in affective dispositions rather than objective environmental conditions—with direct implications for how self-report surveys are designed and interpreted in research and practice.

**Supplementary Information:**

The online version contains supplementary material available at 10.1038/s41598-026-55924-9.

## Introduction

Work environment conditions (e.g., job demands, role clarity, and work relationships) are consistently linked to employee well-being^1^. A recurring challenge in this literature, however, is that studies relying on employee self-report cannot easily distinguish between the environment as it objectively exists and the environment as it is perceived and reported^2–4^. Self-reported work environment data are at least partly shaped by employees’ own emotional dispositions^5^. Employees higher in negative affect tend to report their work environment more negatively regardless of its objective features, while those higher in positive affect tend to report it more favourably—both inflating observed associations between environmental conditions and well-being outcomes^6,7^. The present study used a large, stratified Danish sample to quantify the extent to which individual differences in affect explain the relationships between work environment perceptions and employee well-being, both cross-sectionally and longitudinally.

### Work environment and employee well-being

The work environment is here understood broadly as the psychosocial conditions under which employees operate, including job demands (quantitative and emotional), resource-based factors (role clarity and employee influence), and interpersonal relationships (teamwork and leadership quality)^8^. Employee well-being refers to how workers feel over time in relation to their workplace, operationalised here as perceived job stress, self-rated health, and job satisfaction. Each of these work environment dimensions has been linked to employee well-being: demands are consistently associated with elevated stress and reduced satisfaction^9–11^, resource-based factors with reduced stress and higher well-being^12–14^, and positive interpersonal relationships, particularly leadership quality, with employee satisfaction^15–17^.

### Negative affect and positive affect at work

Not every employee reacts in the same way to the same work environment—some are more content, while others are more stressed. Two fundamental affective dimensions, negative affect (NA) and positive affect (PA), have been shown to play a significant role across all domains of well-being^18,19^. NA is characterised by a heightened susceptibility to negative experiences and stressful environments, including workplace stress^20–24^, and extends to physical and mental health outcomes^25–27^. Higher NA has also been associated with greater perceived interpersonal conflict at work^28^ and increased demands to regulate emotions in social interactions^29^, suggesting a particular sensitivity to the social and affective features of the work environment. PA, in contrast, underlies outgoing, energetic behaviour and is associated with job satisfaction, general happiness, and overall life satisfaction^30,31^, meaning individuals high in PA are more likely to perceive their work environments favourably, regardless of objective conditions.

NA and PA are not simply outcomes of the work environment—they also shape how employees perceive and report it. There are good reasons to treat them as stable individual characteristics rather than as state responses to working conditions. For one, genetic factors account for roughly half the variance in most individual differences, including affect^32^, suggesting a dispositional baseline that is largely independent of any particular work context. Beyond that, environmental experiences tend to have only small and transient effects on affective dispositions^33^, and even discrete negative events account for at most 19% of within-person affective variance at the momentary level^34^. Consistent with this, self-reported NA and PA show reasonably stable test-retest reliability over a year^35^, in line with their characterisation as trait-like tendencies. Taken together, this supports treating NA and PA as individual-difference controls in the present study, rather than as outcomes the work environment might be producing.

### Research question

The present study aimed to quantify the extent to which negative and positive affect explain both cross-sectional and longitudinal relationships between the work environment and employee well-being. The research question was as follows: How much of the relationship between the work environment (i.e., quantitative and emotional demands, job role clarity, employee influence, teamwork, and leadership) and employee well-being (i.e., stress, health, and job satisfaction) can be explained by individual differences in emotional experiences (i.e., negative affect and positive affect)?

## Method

### Participants and procedure

The DPQ was distributed to a stratified, mostly representative, sample of 8,958 employed individuals in Denmark, representing the Danish labour market. Data from this survey have been used in prior studies^36–39^, and the sampling procedure is described in detail elsewhere^8,40^. The present study was restricted to employees with stable labour market attachment to ensure sufficient exposure to the work environment. Participants were excluded if they were unemployed at baseline, had overlapping age-group coding boundaries, or had missing data exceeding 80% of questionnaire items prior to multiple imputation. At baseline (Wave T1; 48.4% response rate), *N* = 3,970 remained after applying these criteria. The sample was equally distributed across 14 job groups, including office work, healthcare and social services, education, manufacturing, law enforcement, and business management (range = 5–9%). Women constituted 47% of the sample; age distribution was: 6% aged 18–34, 19% aged 35–44, 32% aged 45–54, and 43% over 54. Additionally, 26% held a high school diploma, 15% a short-cycle tertiary education, 27% a medium-cycle tertiary education, and 19% a long-cycle tertiary education. At six-month follow-up (Wave T2), 2,375 participants responded. 

### Instruments 

[See appendix table S4 for complete overview of the questionnaire and constructs used.]

#### Quantitative demands/work environment

The quantitative demands scale in the DPQ was used to estimate workload. The scale consists of four items with questions like “How often is it the case that you do not have time to complete all your work tasks?” and “How often do you have deadlines that are hard to meet?” using a 5-point Likert scale ranging from “Never/almost never” (1) to “Always” (5). The reliability (Cronbach’s alpha) is reportedly 0.84 with a test-retest reliability of 0.78^8^.

#### Emotional demands/work environment

The emotional demands scale in the DPQ consists of four items with questions like “Are you placed in emotionally demanding situations at work?” and “Do you have to deal with relationships at work that are emotionally challenging?” using a 5-point Likert scale ranging from “To a very small extent” (1) to “To a very large extent” (5). The Cronbach’s alpha was 0.83 with a test-retest reliability of 0.74^8^.

#### Job role clarity/work environment

The role clarity scale in the DPQ consists of four items. Questions such as “Are there any conflicting demands in your work?” and “Does your job involve tasks that conflict with your personal values?” were answered using a 5-point Likert scale ranging from “To a very small extent” (1) to “To a very large extent” (5). The Cronbach’s alpha documented for this scale is 0.81 with a test-retest reliability of 0.73^8^.

#### Employee influence/work environment

The influence at work scale in the DPQ consists of four items such as “Do you have any influence on how you carry out your work tasks?” and “Do you have sufficient authority to deal with the responsibilities you have in your work?” using a 5-point Likert scale ranging from “To a very small extent” (1) to “To a very large extent” (5). The Cronbach’s alpha documented for this scale is 0.87, with a test-retest reliability of 0.78^8^.

#### Teamwork/work environment

This scale in the DPQ was used to capture the sense of cooperation measured by four items, such as “Do you and your colleagues work well together when problems emerge which require cooperation among you?” and “Is there a sense of community and cohesion between you and your colleagues?” using a 5-point Likert scale ranging from “To a very small extent” (1) to “To a very large extent” (5). The Cronbach’s alpha documented for this scale is 0.82 with a test-retest reliability of 0.75^8^.

#### Leadership/work environment

The quality of leadership scale in the DPQ consists of four items with questions such as “Is your immediate supervisor good at motivating the employees?” and “Is your immediate supervisor good at communicating clear goals for the work of you and your colleagues?” using a 5-point Likert scale ranging from “To a very small extent” (1) to “To a very large extent” (5). The Cronbach’s alpha documented for this scale is 0.91 with a test-retest reliability of 0.87^8^.

#### Job stress/employee well-being

Perceived job stress was measured with a single item: “How often have you felt stressed in the last two weeks?“. This question was answered using a Likert scale ranging from (1) Never—(5) Always. This item has a reported test-retest reliability of 0.75^8^.

#### Health/employee well-being

Self-rated health was measured with a single item: “Overall, how do you perceive your health?“. This question was answered using a Likert scale ranging from (1) Very low—(5) Very high.

#### Job satisfaction/employee well-being

One item was used to operationalise how much the employee likes the job: “Overall, how satisfied are you with your job?” 0 denotes the lowest possible level of job satisfaction, and 10 denotes the highest possible level. The test-retest reliability for this item is 0.77^8^.

#### Negative affect/control variable

A brief measure of negative affect was assessed with four items on a 6-point Likert scale from “At no time” (1) to “All the time” (6): “How much of the time in the last two weeks have you felt calm and relaxed?“, “.been sad or upset?“, “.had lower self-confidence?“, and “.had a bad conscience or feelings of guilt?”^41^. These items bear resemblance to the PANAS negative affect subscale (see Table 1 and ref^42^), which has been extensively validated in workplace environments^7,35,43–45^. At the six-month follow-up, the scale showed a test-retest reliability of *r* = 0.63, consistent with the 12-month PANAS benchmark of *r* > 0.60^45^.

#### Positive affect/control variable

A brief measure of positive affect was captured with three items using the same 6-point Likert scale^46^: “How much of the time in the last two weeks have you felt quiet or reserved?“, “.been active and energetic?“, and “.been happy and in good mood?” (see Table 1). At the six-month follow-up, the scale showed a test-retest reliability of *r* = 0.61, consistent with the reported 12-month PANAS benchmark of *r* = 0.63^45^.

#### Note on recall window and trait interpretation

The two-week recall window is worth addressing, as it raises the question of whether the affect scales are capturing stable dispositions or more transient emotional states. According to Robinson and Clore’s accessibility model^47^, affect reports covering periods beyond a few days can no longer rely on episodic memory of specific emotional events; respondents instead draw on semantic memory, that is, stable, trait-consistent beliefs about their own emotional dispositions. Consistent with this, self-reported affect intensity tends to increase as the recall window lengthens^48^, and retrospective ratings over longer windows are systematically biased toward trait-consistent representations, particularly for negative affect^49^. The observed test-retest reliabilities (*r* = 0.63 and 0.61 at six months) are therefore best interpreted as conservative lower bounds on trait-level stability, and any residual state variance in the scores would attenuate rather than inflate the reported variance-reduction estimates. If anything, the present findings likely underestimate the true contribution of stable affect.

### Statistical analysis

Assumptions of normality and linearity, including missing values and outliers, were evaluated prior to analyses^50^. Follow-up data were used to calculate test-retest reliabilities for negative affect and positive affect scales.

Partial correlational analyses were conducted to address the research question. We calculated correlations for all study variables while controlling for the overlapping variance of positive and negative affect. We also calculated correlations between baseline (Wave T1) work environment and employee well-being six months later (Wave T2) and quantified the difference in explained variance between the zero-order and partial correlations by calculating the percentage reduction (see ref. 6 and ref. 43). The explained variance reduction rate indicates the percentage of the work environment conditions-outcomes relationship that is accounted for by the two individual characteristics. We used Chen and Spector’s formula^43^ to calculate the explained variance reduction rate:

$$\text{Percentage reduction rate in } R^{2}=\frac{\left(\:{R}_{zero-order\:-\:}^{2}{R}_{partial}^{2}\right)}{{R}_{zero-order\:\:}^{2}}\:$$

We also calculated the reduction rate of BIC values between the zero-order and partial correlations to address model complexity; larger reduction rates indicate that negative and positive affect improve model fit substantially relative to the increase in complexity^51^. To interpret correlations, we adhered to effect size guidelines^52^, where *r* < 0.10 is considered trivial (< 1%) and *r* > 0.30 strong, in most individual difference research. Furthermore, power analysis (80% power) on multiple correlations (regressions) indicated that at a sample size of *N* = 1,200, an effect size of *r* = 0.10 is significant at *p* < 0.01. Last, seeing the large number of estimates (11 × 10 variables) in the present study, we applied Bonferroni-correction to the significance testing.

### Ethical consideration

The dataset was collected and managed by the Danish National Research Centre for the Working Environment (NRCWE) in accordance with the EU General Data Protection Regulation (GDPR). All participants provided informed consent prior to completing the questionnaire. The data were fully anonymised, and discretion considerations were applied throughout to prevent the identification of individual persons in the data material. The first author accessed the dataset through an employment affiliation with NRCWE; data ownership remains with NRCWE and the dataset is not publicly available.

In Sweden, formal ethical review under the *Act Concerning the Ethical Review of Research Involving Humans* (SFS 2003:460) is not required for observational, self-report research that does not involve sensitive personal data, physical intervention, or methods that risk harm to participants. The present study met none of these criteria. The study was therefore conducted in accordance with the ethical guidelines of Lund University and the principles of the Declaration of Helsinki.

## Results

In preparatory analyses, Little’s MCAR test indicated that the observed pattern of missing values was random (*χ²*(*df* = 7741) = 4671, *p* = 1). When data are missing at random and the proportion of missing values is small (< 5%), the choice of imputation method has little bearing on the results. Therefore, we handled missing data with Multiple Imputation by Chained Equations (MICE) to preserve the natural variability in the data^53^. Univariate normality was confirmed with skew and kurtosis values, and linearity was confirmed via scatterplots. All descriptive statistics at the first survey measurement T1 are reported in Table 2. All variable reliabilities were satisfactory, *α* > 0.76.

The cross-sectional correlations at T1 are presented in Table 2. Overall, correlations between work environment and employee well-being ranged from moderate (*r* ≈ 0.20) to high (*r* ≈ 0.50), with notable associations between quantitative demands and job stress (*r* = 0.45) and job satisfaction and leadership (*r* = 0.47). Both NA and PA correlated moderately with work environment variables 1–6 (*r* mean = 0.28) and more strongly with employee well-being variables 7–9 (*r* mean = 0.45). Longitudinal zero-order correlations between T1 work environment and T2 employee well-being are reported in the Appendix Tables, with broadly similar patterns.

### Work environment and longitudinal employee well-being

Partial correlations controlling for baseline NA and PA are reported both cross-sectionally for T1 and longitudinally with T2 well-being measures six months later (Table 3). Individual differences in affect accounted for substantial portions of the work environment-well-being associations. On average, NA and PA explained 78% of the cross-sectional variance for job stress, 91% for health, and 49% for job satisfaction. Longitudinally, these figures were remarkably similar at 79%, 89%, and 57%, respectively. In several cases, individual differences accounted for nearly all of the observed association, such as between job role clarity and health (100% T1; 94% T2) or employee influence and job stress (97% T1; 92% T2). Across all conditions and outcomes, affect explained an average of 73% cross-sectionally and 75% longitudinally. The inclusion of NA and PA also improved model fit (*Δ*BIC rate) by 5.7% to 12.7% in the cross-sectional models and 3.5% to 5.8% in the longitudinal models.

As a robustness check, two representative relationships were estimated using a structural after measurement (SAM) approach to account for measurement error^54^. When treating NA and PA as latent variables, the dispositional signal was somewhat stronger than in the observed partial correlations. The quantitative demands → job stress path reduced from *β* = 0.487 to *β* = 0.353 after controlling for latent NA and PA (47% variance reduction, vs. 33% in the observed partial correlations), and the leadership → job satisfaction path reduced from *β* = 0.486 to β = 0.337 (52% variance reduction, vs. 35% observed). These results are consistent with the interpretation that the partial correlation estimates in Table 3 represent conservative lower bounds on the true explanatory contribution of affect.

## Discussion

In a stratified national sample of 3,970 employees, individual differences in affect consistently and substantially reduced associations between perceived work environment and well-being. The findings extend the dispositional inflation literature^6,43^ in a few notable ways. For one, prior work has largely examined dispositional inflation in narrow, domain-specific contexts^55,56^, whereas the present findings suggest the pattern generalises broadly across six work environment dimensions and three well-being outcomes. The pattern also held both cross-sectionally and longitudinally, suggesting dispositional inflation is robust to temporal spacing and unlikely to reflect a single-occasion measurement artifact^57^. Notably, inflation was not uniform—associations with health perception were more strongly attenuated than those with job satisfaction, consistent with the observability principle^58^, which suggests that subjective, internally experienced outcomes are more susceptible to affective influence than evaluations of tangible job features.

It could be argued that affective characteristics are shaped by the work environment rather than the other way around, but the work-specific genetic evidence does not readily support this. NA and PA mediate approximately 45% of the genetic variance in job satisfaction, outperforming personality as a mediating framework^59^, and this genetic mediation remains stable across three measurement waves spanning early to mid-adulthood^60^. More tellingly, genetic influences extend to perceived work stress and downstream health outcomes, with the mediated pathways themselves being genetic^61^, suggesting that what appears to be an environmental effect on health is, in substantial part, dispositional in origin. The present pattern of results is therefore more consistently explained by stable affective dispositions filtering perceptions of the work environment than by the environment producing lasting changes in affect.

## Limitations

Both working conditions and well-being were assessed via self-report, meaning the findings cannot be triangulated against independent measures of the work environment. This makes it difficult to determine whether the residual variance—the portion not explained by NA and PA—reflects objective environmental conditions, other unmeasured individual differences, or both. Future studies incorporating observer-rated or administrative measures alongside self-report would allow this residual to be more precisely attributed.

The longitudinal design also does not allow us to establish causal directionality or rule out reciprocal effects between work environment perceptions and employee well-being.

Operationalisation of affect was also constrained by the pre-set items available in the Danish Psychosocial Questionnaire, and the brevity of the resulting scales means they cannot fully capture the depth and breadth of individual differences in emotional dispositions. As the SAM robustness check confirmed, the present estimates are best regarded as conservative lower bounds — more psychometrically differentiated measures would likely yield larger estimates.

The NA and PA scales also showed a stronger negative intercorrelation (*r* = − 0.74) than typically reported for PANAS (*r* ≈ − 0.24;^62^, suggesting some construct overlap in this sample. Variance inflation factors were nonetheless low (VIF < 2), indicating that multicollinearity did not distort the partial correlation estimates and that each dimension contributed independently to the observed variance reduction.

Finally, the sample was treated as a single generalised employee group. Subgroup analyses by gender confirmed the pattern was consistent across groups, which aligns with the broader individual differences literature showing minimal demographic effects^63^.

### Recommendations and concluding thoughts

Based on these findings, we recommend that future studies using self-report to estimate the effect of working conditions on employee well-being routinely include measures of affective disposition as control variables, provided the research goal is to isolate environmental effects. If the aim is environmental attribution, the more fundamental solution is stronger measurement design — triangulating self-report with observer-rated or administrative measures, or using items less susceptible to subjective interpretation. Where the research interest lies in affect itself, NA and PA should be treated as focal variables rather than noise to be removed^64^.

For practitioners, the implication is more interpretive than methodological. Organisations routinely use engagement and well-being surveys as diagnostic tools, but aggregate scores may reflect the affective composition of a team as much as the quality of its environment. Without sufficient expertise in survey design and interpretation, such instruments risk producing misleading signals that drive misguided interventions. The solution is not to collect individual-level affective data, which carries real ethical risks—including potential misuse in hiring or performance appraisal. Individual differences in affect are better understood as signals of person-environment fit^65^, best addressed through shared organisational responsibility for role design rather than individual classification.

Well-being surveys are often treated as windows onto the work environment. The present findings suggest they may also function, in part, as mirrors — reflecting who is answering as much as what the environment is actually like. Accounting for individual differences is not just a statistical nicety. It is a precondition for interpreting what such surveys actually tell us.

**Table 1:** Positive and negative affect in the present study.

DPQ	PANAS
Positive affect	
Quiet or reserved (−)	Enthusiastic
Active and energetic	Active and alert
Good mood	Excite and inspired
Negative affect	
Calm and relaxed (−)	Nervous
Sad and upset	Upset
Low self-confidence	Strong
Feelings of guilt	Guilty

See Appendix Table S4 for the complete study variable items.

**Table 2:** Descriptive statistics and study correlations between work environment (1–6) T1 and employee well-being (7–9) T1.

Variables	M	SD	α	Skew	Kurtosis	1	2	3	4	5	6	7	8	9	10
(1) Quantitative demands	8.91	3.16	0.84	− 0.15	− 0.3	–									
(2) Emotional demands	7.17	3.67	0.83	0.29	− 0.59	0.34	–								
(3) Job role clarity	12.33	2.68	0.82	− 0.47	0.57	− 0.24	− 0.11	–							
(4) Employee influence	11.28	3.44	0.87	− 0.48	0.06	0.01	− 0.07	0.35	–						
(5) Teamwork	11.52	2.85	0.82	− 0.47	0.61	− 0.19	− 0.09	0.42	0.33	–					
(6) Leadership	10.06	3.72	0.91	− 0.4	− 0.1	− 0.20	− 0.13	0.45	0.32	0.49	–				
(7) Job stress	2.52	1.05	–	0.24	− 0.61	0.45	0.27	− 0.22	− 0.13	− 0.23	− 0.23	–			
(8) Health	3.52	0.91	–	− 0.22	− 0.22	− 0.07	− 0.08	0.15	0.20	0.20	0.17	− 0.26	–		
(9) Job satisfaction	7.30	2.06	–	− 1.02	0.92	− 0.20	− 0.19	0.46	0.50	0.44	0.47	− 0.30	0.26	–	
(10) Negative affect	5.55	3.4	0.80	1.23	1.36	0.29	0.24	− 0.34	− 0.23	− 0.31	− 0.26	0.55	− 0.33	− 0.44	–
11. Positive affect	12.00	2.44	0.76	-1.17	1.35	− 0.21	− 0.17	0.35	0.28	0.35	0.31	− 0.47	0.42	0.48	− 0.74

*N* = 3970. *M* = Mean. *SD* = Standard deviation. *α* = Cronbach’s alpha. Variables 1–6 are work environment conditions; quantitative demands, emotional demands, job role clarity, employee influence, teamwork, leadership (scaled 1–17); In grey are employee well-being variables (7, 8, 9); Job stress and Health (scaled 1–5); Job Satisfaction (scaled 0–10); Negative affect (scaled 1–21); Positive affect (scaled 1–16). After Bonferroni-correction *r* > = 0.10 was significant at *p* < 0.00027. See Appendix for correlational table between work conditions T1 and outcomes T2.

**Table 3:** Relationships between work environment measures Wave T1 and three employee well-being measures in waves T1 and T2.

	T1 to T1				T1 to T2			
	Zero- order (*r*)	Partial (*r*)	% PANAS	ΔBIC rate	Zero-order (*r*)	Partial (*r*)	% PANAS	ΔBIC rate
Job stress								
Quant. Demands sdemands	0.45	0.37	33%	11.3%	0.36	0.27	44%	4.3%
Emo. Demands	0.27	0.18	58%	11.9%	0.25	0.16	57%	5.2%
Job role clarity	− 0.22	− 0.03	98%	11.6%	− 0.19	− 0.04	96%	5.2%
Employee influence	− 0.13	0.02	97%	12.7%	− 0.13	− 0.04	92%	5.8%
Teamwork	− 0.23	− 0.06	94%	11.6%	− 0.19	− 0.05	94%	5.3%
Leadership quality	− 0.23	− 0.09	85%	11.8%	− 0.17	− 0.06	88%	5.5%
Mean average	0.26	0.12	78%	11.8%	0.21	0.10	79%	5.2%
Health								
Quant. Demands	− 0.07	0.03	82%	6.7%	− 0.11	− 0.02	97%	4.6%
Emo. Demands	− 0.08	− 0.01	99%	6.6%	− 0.10	− 0.03	93%	4.8%
Job role clarity	0.15	0.00	100%	6.1%	0.19	0.05	94%	3.9%
Employee influence	0.20	0.10	77%	5.8%	0.22	0.12	69%	4.0%
Teamwork	0.20	0.06	92%	5.7%	0.20	0.07	87%	3.8%
Leadership quality	0.17	0.04	94%	5.9%	0.16	0.04	94%	4.2%
Mean average	0.15	0.04	91%	6.1%	0.16	0.05	89%	4.2%
Job satisfaction								
Quant. Demands	− 0.20	− 0.10	78%	8.6%	− 0.18	− 0.08	82%	5.3%
Emo. Demands	− 0.19	− 0.11	70%	8.9%	− 0.17	− 0.09	72%	5.6%
Job role clarity	0.46	0.34	44%	6.3%	0.36	0.23	58%	3.5%
Employee influence	0.50	0.43	25%	7.7%	0.42	0.35	31%	4.2%
Teamwork	0.44	0.33	45%	6.5%	0.36	0.24	54%	3.7%
Leadership quality	0.47	0.38	35%	7.1%	0.39	0.29	43%	3.9%
Mean average	0.38	0.28	49%	7.5%	0.31	0.21	57%	4.4%
Total average	0.26	0.15	73%	8.5%	0.23	0.12	75%	4.6%

Underscores signify employee well-being measures. Zero-order correlation = bivariate Pearson’s correlations at T1; Partial correlation = Zero-order correlation controlled for negative affect and positive affect at T1; T1 = baseline *N* = 3,970, T2 = 6 months follow-up *N* = 2375; T2% Ind. Differences are the explained variance between T1 work conditions and T2 employee outcomes. After Bonferroni-correction *r* > = 0.10 was significant at *p* < 0.00027; % Individual Differences = *R*^*2*^ reduction rate (%) between zero-order and partial correlations; *Δ*BIC rate = % improvement in model fit between zero-order and partial correlations.

## Supplementary Information

Below is the link to the electronic supplementary material.

Supplementary Material 1

Supplementary Material 2

Supplementary Material 3

## Data Availability

This study’s design and its analysis were not preregistered, and materials are not available because they are not owned by the authors. The dataset is owned by Danish National Research Centre for the Working Environment (NRCWE) and is not publicly accessible. The first author had access to the dataset through an employment affiliation.

## Code availability

The code that produced these results is available in supplemental material.
