# Supplementary material for: Individual differences in affect: explaining work environment perceptions and later wellbeing
Source: Sci Rep. 2026 Jun 11;16:18178. doi: 10.1038/s41598-026-55924-9 (PMC13260830; doi:10.1038/s41598-026-55924-9)
Supplement: Supplementary file 1 — Supplementary Material 1 [file 41598_2026_55924_MOESM1_ESM.pdf]

```
#
=======================================================================
=======
# ANALYSIS SCRIPT
# "Individual Differences in Employees: How Affect Shapes Work Environment
#  Perceptions and Longitudinal Wellbeing"
#
=======================================================================
=======
# Outputs (commented out — uncomment to save):
#   Table2.csv   — Descriptives + T1 correlation matrix
#   Table3.csv   — Zero-order r, partial r, % PANAS reduction, ΔBIC rate
#   TableS1.csv  — T1 work environment × T2 well-being correlations
#   TableS2.csv  — T2 well-being × T2 NA/PA correlations
#   TableS3.csv  — NA/PA item-level correlations T1 & T2
#
=======================================================================
=======
# Note: Raw data are not publicly available (registry restrictions).
#       This script is provided for transparency and reproducibility of
#       analytical decisions.
#
=======================================================================
=======


library(psych)
library(ppcor)
library(tidyverse)


# — Load data
```

```
data <- read.csv("/FINALDATA.csv")


#
=============================================================================
=======

# CRONBACH'S ALPHA — All multi-item scales

#
=============================================================================
=======


alpha_results <- list(

  QuantDemands = psych::alpha(data[, c("RA15_Q11_01_SAMLET",
"RA15_Q11_02_SAMLET",

                          "RA15_Q11_04_SAMLET", "RA15_Q11_05_SAMLET")],

                    check.keys = TRUE)$total$raw_alpha,

  EmoDemands   = psych::alpha(data[, c("RA15_Q11_11_SAMLET",
"RA15_Q11_12_SAMLET",

                          "RA15_Q11_13_SAMLET", "RA15_Q11_14_SAMLET")],

                    check.keys = TRUE)$total$raw_alpha,

  RoleClarity  = psych::alpha(data[, c("RA15_Q10_06", "RA15_Q10_07",

                          "RA15_Q10_09", "RA15_Q10_10")],

                    check.keys = TRUE)$total$raw_alpha,

  Influence    = psych::alpha(data[, c("RA15_Q09_10", "RA15_Q09_11",

                          "RA15_Q09_12", "RA15_Q09_13")],

                    check.keys = TRUE)$total$raw_alpha,

  Teamwork     = psych::alpha(data[, c("RA15_Q14_03", "RA15_Q14_04",

                          "RA15_Q14_09", "RA15_Q14_11")],

                    check.keys = TRUE)$total$raw_alpha,

  Leadership   = psych::alpha(data[, c("RA15_Q21_05", "RA15_Q21_07",

                          "RA15_Q21_11", "RA15_Q21_13")],
```

```
                    check.keys = TRUE)$total$raw_alpha,
  NA_T1      = psych::alpha(data[, c("RA15_Q31_02", "RA15_Q31_06",
                          "RA15_Q31_09", "RA15_Q31_10")],
                    check.keys = TRUE)$total$raw_alpha,
  PA_T1      = psych::alpha(data[, c("RA15_Q31_01", "RA15_Q31_03",
                          "RA15_Q31_15")],
                    check.keys = TRUE)$total$raw_alpha,
  NA_T2      = psych::alpha(data[, c("RA15_Q31_02_FU", "RA15_Q31_06_FU",
                          "RA15_Q31_09_FU", "RA15_Q31_10_FU")],
                    check.keys = TRUE)$total$raw_alpha,
  PA_T2      = psych::alpha(data[, c("RA15_Q31_01_FU", "RA15_Q31_03_FU",
                          "RA15_Q31_15_FU")],
                    check.keys = TRUE)$total$raw_alpha
)

# Print alpha summary
alpha_df <- data.frame(
  Scale = names(alpha_results),
  Alpha = round(unlist(alpha_results), 2)
)
print(alpha_df)

# ── Format alpha for tables ─────────────────────────────────────────
fmt_alpha <- function(x) sprintf("%.2f", x)

# ── Standardize variables (z-scores) ────────────────────────────────
variables_to_standardize <- c(
```

```
  "RA15_QUANTITATIVEDEMANDS", "RA15_EMOTIONALDEMANDS",
  "RA15_ROLECLARITY",        "RA15_INFLUENCE",
  "RA15_COOPERATION",        "RA15_LEADQUAL",
  "T1_NA",  "T1_PA",
  "T1_STRESS",  "T2_STRESS",
  "T1_HEALTH",  "T2_HEALTH",
  "T1_JOBSAT",  "T2_JOBSAT"
)

for (var in variables_to_standardize) {
  data[[paste0(var, "_z")]] <- as.numeric(scale(data[[var]]))
}

# ── Helper: format r (strip leading zero) ──────────────────────────────
fmt_r     <- function(x) sub("^(-?)0\\.", "\\1.", sprintf("%.2f", x))
fmt_r_tbl <- function(x) ifelse(is.na(x), "",
                         sub("^(-?)0\\.", "\\1.", sprintf("%.2f", x)))

#
=========================================================================
=======
# TABLE 2 — Descriptives + T1 Correlation Matrix
#
=========================================================================
=======

table2_vars <- c(
  "RA15_QUANTITATIVEDEMANDS", "RA15_EMOTIONALDEMANDS",
  "RA15_ROLECLARITY",        "RA15_INFLUENCE",
```

```
  "RA15_COOPERATION",      "RA15_LEADQUAL",
  "T1_STRESS", "T1_HEALTH",  "T1_JOBSAT",
  "T1_NA",    "T1_PA"
)

table2_labels <- c(
  "1. Quantitative Demands", "2. Emotional Demands",
  "3. Job Role Clarity",    "4. Employee Influence",
  "5. Teamwork",          "6. Leadership",
  "7. Job Stress",         "8. Health",
  "9. Job Satisfaction",    "10. Negative Affect",
  "11. Positive Affect"
)

alphas2 <- c(
  fmt_alpha(alpha_results$QuantDemands),
  fmt_alpha(alpha_results$EmoDemands),
  fmt_alpha(alpha_results$RoleClarity),
  fmt_alpha(alpha_results$Influence),
  fmt_alpha(alpha_results$Teamwork),
  fmt_alpha(alpha_results$Leadership),
  "—", "—", "—",   # single-item: Stress, Health, Job Satisfaction
  fmt_alpha(alpha_results$NA_T1),
  fmt_alpha(alpha_results$PA_T1)
)

desc2 <- psych::describe(data[, table2_vars])[, c("mean", "sd", "skew", "kurtosis")]
desc2 <- round(desc2, 2)
```

```
cor2      <- cor(data[, table2_vars], use = "pairwise.complete.obs")

cor2_lower <- cor2

cor2_lower[upper.tri(cor2_lower, diag = TRUE)] <- NA

cor2_disp  <- apply(round(cor2_lower, 2), c(1, 2), fmt_r_tbl)


table2 <- data.frame(

  Variable = table2_labels,

  M       = desc2$mean,

  SD      = desc2$sd,

  Alpha    = alphas2,

  Skew     = desc2$skew,

  Kurtosis = desc2$kurtosis,

  cor2_disp,

  stringsAsFactors = FALSE, check.names = FALSE

)

colnames(table2)[7:17] <- as.character(1:11)


print(table2)


write.table(table2, "H:/Publications/OneDrive_2026-02-13/Speciale
Paper/Analyses/Results/Table2.csv", sep = ",", row.names = FALSE, quote = TRUE)


#
=========================================================================
=======

# TABLE 3 — Zero-order r, Partial r, % PANAS Reduction, ΔBIC Rate

# Logic per cell:

#   Model 1: outcome ~ predictor                   (zero-order)
```

```
#   Model 0: outcome ~ T1_NA_z + T1_PA_z           (PANAS-only baseline)
#   Model 2: outcome ~ predictor + T1_NA_z + T1_PA_z (full model)
#   % PANAS = ((R²_M1 - (R²_M2 - R²_M0)) / R²_M1) * 100
#   ΔBIC rate = ((BIC_M1 - BIC_M2) / |BIC_M1|) * 100
#
===============================================================================

get_table3_row <- function(predictor, outcome_T1, outcome_T2,
                           label_predictor, label_outcome, data) {

  covariates <- c("T1_NA_z", "T1_PA_z")

  compute_cell <- function(outcome) {
    d <- data[complete.cases(data[, c(outcome, predictor, covariates)]), ]

    m1 <- lm(as.formula(paste(outcome, "~", predictor)), data = d)
    m2 <- lm(as.formula(paste(outcome, "~", predictor, "+ T1_NA_z + T1_PA_z")), data = d)

    r_zero    <- cor(d[[outcome]], d[[predictor]], use = "complete.obs")
    pcor_res  <- pcor.test(d[[outcome]], d[[predictor]], d[, covariates])
    r_partial <- pcor_res$estimate

    r2_m1 <- summary(m1)$r.squared
    r2_m2 <- summary(m2)$r.squared

    pct_panas     <- ifelse(r_zero != 0,
                       ((r_zero^2 - r_partial^2) / r_zero^2) * 100, NA)
    delta_bic_rate <- ((BIC(m1) - BIC(m2)) / abs(BIC(m1))) * 100
```

```
  list(
    r0_fmt   = fmt_r(r_zero),
    rp_fmt   = fmt_r(r_partial),
    red_fmt  = paste0(round(pct_panas), "%"),
    dbic_fmt = paste0(round(delta_bic_rate, 1), "%"),
    r0_num   = abs(r_zero),
    rp_num   = abs(r_partial),
    red_num  = pct_panas,
    dbic_num = delta_bic_rate
  )
}

T1 <- compute_cell(outcome_T1)
T2 <- compute_cell(outcome_T2)

data.frame(
  Outcome            = label_outcome,
  Predictor          = label_predictor,
  `Zero-order r (T1)` = T1$r0_fmt,
  `Partial r (T1)`    = T1$rp_fmt,
  `% PANAS (T1)`      = T1$red_fmt,
  `ΔBIC rate (T1)`    = T1$dbic_fmt,
  `Zero-order r (T2)` = T2$r0_fmt,
  `Partial r (T2)`    = T2$rp_fmt,
  `% PANAS (T2)`      = T2$red_fmt,
  `ΔBIC rate (T2)`    = T2$dbic_fmt,
  r0_T1   = T1$r0_num,  rp_T1   = T1$rp_num,
```

```
  red_T1  = T1$red_num, dbic_T1 = T1$dbic_num,
  r0_T2   = T2$r0_num,  rp_T2   = T2$rp_num,
  red_T2  = T2$red_num, dbic_T2 = T2$dbic_num,
  stringsAsFactors = FALSE, check.names = FALSE
 )
}

# — Define predictors and outcomes ———————————————————————

predictors <- list(
 c("RA15_QUANTITATIVEDEMANDS_z", "Quantitative Demands"),
 c("RA15_EMOTIONALDEMANDS_z",    "Emotional Demands"),
 c("RA15_ROLECLARITY_z",        "Job Role Clarity"),
 c("RA15_INFLUENCE_z",          "Employee Influence"),
 c("RA15_COOPERATION_z",        "Teamwork"),
 c("RA15_LEADQUAL_z",           "Leadership Quality")
)

outcomes <- list(
 c("T1_STRESS_z",  "T2_STRESS_z",  "Job Stress"),
 c("T1_HEALTH_z",  "T2_HEALTH_z",  "Health"),
 c("T1_JOBSAT_z",  "T2_JOBSAT_z",  "Job Satisfaction")
)

# — Run all combinations ———————————————————————————————

numeric_cols <- c("r0_T1","rp_T1","red_T1","dbic_T1",
         "r0_T2","rp_T2","red_T2","dbic_T2")
```

```r
raw_rows <- do.call(rbind, lapply(outcomes, function(out) {
  do.call(rbind, lapply(predictors, function(pred) {
    get_table3_row(pred[1], out[1], out[2], pred[2], out[3], data)
  }))
}))

# —— Helper: average row ——————————————————————————————————————————

make_avg_row <- function(df, outcome_label, pred_label = "    Mean Average") {
  avgs <- colMeans(df[, numeric_cols], na.rm = TRUE)
  data.frame(
    Outcome           = outcome_label,
    Predictor         = pred_label,
    `Zero-order r (T1)` = fmt_r(avgs["r0_T1"]),
    `Partial r (T1)`    = fmt_r(avgs["rp_T1"]),
    `% PANAS (T1)`      = paste0(round(avgs["red_T1"]), "%"),
    `ΔBIC rate (T1)`    = paste0(round(avgs["dbic_T1"], 1), "%"),
    `Zero-order r (T2)` = fmt_r(avgs["r0_T2"]),
    `Partial r (T2)`    = fmt_r(avgs["rp_T2"]),
    `% PANAS (T2)`      = paste0(round(avgs["red_T2"]), "%"),
    `ΔBIC rate (T2)`    = paste0(round(avgs["dbic_T2"], 1), "%"),
    r0_T1   = avgs["r0_T1"],  rp_T1   = avgs["rp_T1"],
    red_T1  = avgs["red_T1"], dbic_T1 = avgs["dbic_T1"],
    r0_T2   = avgs["r0_T2"],  rp_T2   = avgs["rp_T2"],
    red_T2  = avgs["red_T2"], dbic_T2 = avgs["dbic_T2"],
    stringsAsFactors = FALSE, check.names = FALSE
  )
}
```

```
# ── Assemble with per-outcome averages + total average ──────────────────────

final_rows <- lapply(c("Job Stress", "Health", "Job Satisfaction"), function(lbl) {
  block <- raw_rows[raw_rows$Outcome == lbl, ]
  rbind(block, make_avg_row(block, lbl))
})

total_avg    <- make_avg_row(raw_rows, "", pred_label = "TOTAL AVERAGE")
table3_final <- rbind(do.call(rbind, final_rows), total_avg)[, 1:10]

print(table3_final)
# write.table(table3_final, "H:/Publications/OneDrive_2026-02-13/Speciale Paper/Analyses/Results/Table3.csv", sep = ",", row.names = FALSE, quote = TRUE)

# ===========================================================================
# TABLE S1 — T1 Work Environment × T2 Well-being Correlations
# (restricted to T2 subsample)
# ===========================================================================

tableS1_vars <- c(
  "RA15_QUANTITATIVEDEMANDS", "RA15_EMOTIONALDEMANDS",
  "RA15_ROLECLARITY",       "RA15_INFLUENCE",
  "RA15_COOPERATION",       "RA15_LEADQUAL",
  "T2_STRESS", "T2_HEALTH",  "T2_JOBSAT",
  "T1_NA",     "T1_PA"
```

```
)

tableS1_labels <- c(
  "1. Quantitative Demands T1", "2. Emotional Demands T1",
  "3. Job Role Clarity T1",     "4. Employee Influence T1",
  "5. Teamwork T1",             "6. Leadership T1",
  "7. Job Stress T2",           "8. Health T2",
  "9. Job Satisfaction T2",     "10. Negative Affect T1",
  "11. Positive Affect T1"
)

# Reuse T1 alphas (same scales, T2 subsample)
alphasS1 <- alphas2

# Restrict to participants with T2 data
data_T2 <- data[complete.cases(data[, c("T2_STRESS", "T2_HEALTH", "T2_JOBSAT")]), ]

descS1 <- psych::describe(data_T2[, tableS1_vars])[, c("mean", "sd")]
descS1 <- round(descS1, 2)

corS1      <- cor(data_T2[, tableS1_vars], use = "pairwise.complete.obs")
corS1_lower <- corS1
corS1_lower[upper.tri(corS1_lower, diag = TRUE)] <- NA
corS1_disp  <- apply(round(corS1_lower, 2), c(1, 2), fmt_r_tbl)

tableS1 <- data.frame(
  Variable = tableS1_labels,
  M       = descS1$mean,
```

```
  SD      = descS1$sd,
  Alpha    = alphasS1,
  corS1_disp,
  stringsAsFactors = FALSE, check.names = FALSE
)
colnames(tableS1)[5:15] <- as.character(1:11)

print(tableS1)
# write.table(tableS1, "H:/Publications/OneDrive_2026-02-13/Speciale Paper/Analyses/Results/TableS1.csv", sep = ",", row.names = FALSE, quote = TRUE)

# ===============================================================================
# TABLE S2 — T2 Well-being × T2 NA/PA Correlations
# ===============================================================================

tableS2_vars <- c("T2_STRESS", "T2_HEALTH", "T2_JOBSAT", "T2_NA", "T2_PA")

tableS2_labels <- c(
  "1. Job Stress T2", "2. Health T2", "3. Job Satisfaction T2",
  "4. Negative Affect T2", "5. Positive Affect T2"
)

corS2      <- cor(data_T2[, tableS2_vars], use = "pairwise.complete.obs")
corS2_lower <- corS2
corS2_lower[upper.tri(corS2_lower, diag = TRUE)] <- NA
```

```
corS2_disp  <- apply(round(corS2_lower, 2), c(1, 2), fmt_r_tbl)


tableS2 <- data.frame(
  Variable = tableS2_labels,
  corS2_disp,
  stringsAsFactors = FALSE, check.names = FALSE
)
colnames(tableS2)[2:6] <- as.character(1:5)


print(tableS2)
# write.table(tableS2, "H:/Publications/OneDrive_2026-02-13/Speciale
Paper/Analyses/Results/TableS2.csv", sep = ",", row.names = FALSE, quote = TRUE)


#
==========================================================================
=======
# TABLE S3 — NA/PA Item-level Correlations T1 & T2
#
# Item mapping (Q31 item number = variable suffix):
#   NA items: Q31_02 = Tense (calm/relaxed, reverse-coded)
#          Q31_06 = Sad
#          Q31_09 = Low Confidence
#          Q31_10 = Guilt
#   PA items: Q31_01 = Positive Mood
#          Q31_03 = Energetic
#          Q31_15 = Quiet/reserved (reverse-coded → Talkative)
#
# Note: Reverse-coded items were recoded prior to composite scoring.
#      Item-level correlations reflect scores as stored in the dataset.
```

```
#
==============================================================================
=======

tableS3_vars <- c(
  # NA — T1
  "RA15_Q31_02",    # Tense (calm/relaxed, reverse-coded)
  "RA15_Q31_06",    # Sad
  "RA15_Q31_09",    # Low Confidence
  "RA15_Q31_10",    # Guilt
  # PA — T1
  "RA15_Q31_01",    # Positive Mood
  "RA15_Q31_03",    # Energetic
  "RA15_Q31_15",    # Quiet/reserved (reverse-coded → Talkative)
  # NA — T2
  "RA15_Q31_02_FU", # Tense T2
  "RA15_Q31_06_FU", # Sad T2
  "RA15_Q31_09_FU", # Low Confidence T2
  "RA15_Q31_10_FU", # Guilt T2
  # PA — T2
  "RA15_Q31_01_FU", # Positive Mood T2
  "RA15_Q31_03_FU", # Energetic T2
  "RA15_Q31_15_FU"  # Quiet/reserved T2 (reverse-coded → Talkative)
)

tableS3_labels <- c(
  "1. Tense T1",         "2. Sad T1",
  "3. Low Confidence T1",  "4. Guilt T1",
  "5. Positive Mood T1",   "6. Energetic T1",
```

```
  "7. Talkative T1",
  "8. Tense T2",          "9. Sad T2",
  "10. Low Confidence T2", "11. Guilt T2",
  "12. Positive Mood T2",  "13. Energetic T2",
  "14. Talkative T2"
)

corS3      <- cor(data[, tableS3_vars], use = "pairwise.complete.obs")
corS3_lower <- corS3
corS3_lower[upper.tri(corS3_lower, diag = TRUE)] <- NA
corS3_disp  <- apply(round(corS3_lower, 2), c(1, 2), fmt_r_tbl)

tableS3 <- data.frame(
  Variable = tableS3_labels,
  corS3_disp,
  stringsAsFactors = FALSE, check.names = FALSE
)
colnames(tableS3)[2:15] <- as.character(1:14)

print(tableS3)
# write.table(tableS3, "H:/Publications/OneDrive_2026-02-13/Speciale Paper/Analyses/Results/TableS3.csv", sep = ",", row.names = FALSE, quote = TRUE)

# ===============================================================================
# ROBUSTNESS CHECK — SAM APPROACH
```

```
#
==========================================================================
=======
# SAM separates measurement from structure and provides a latent-variable
# robustness check for the two key relationships:
#   (1) Quantitative Demands  → T1 Stress
#   (2) Leadership Quality    → T1 Job Satisfaction
#
# For each relationship:
#   Model 1 = Zero-order latent association
#   Model 2 = Association controlling for NA_lat and PA_lat
#
==========================================================================
=======

library(lavaan)

# ---------------------------------------------------------------------------
# 1. Quantitative Demands → T1 Stress
# ---------------------------------------------------------------------------

sam_m1_quant <- '
 # Measurement model
 NA_lat       =~ RA15_Q31_02 + RA15_Q31_06 + RA15_Q31_09 + RA15_Q31_10
 PA_lat       =~ RA15_Q31_01 + RA15_Q31_03 + RA15_Q31_15
 QUANTDEM_lat  =~ RA15_Q11_01_SAMLET + RA15_Q11_02_SAMLET +
             RA15_Q11_04_SAMLET + RA15_Q11_05_SAMLET

 # Zero-order structural path
```

```
  T1_STRESS ~ QUANTDEM_lat
'

sam_m2_quant <- '
  # Measurement model
  NA_lat       =~ RA15_Q31_02 + RA15_Q31_06 + RA15_Q31_09 + RA15_Q31_10
  PA_lat       =~ RA15_Q31_01 + RA15_Q31_03 + RA15_Q31_15
  QUANTDEM_lat  =~ RA15_Q11_01_SAMLET + RA15_Q11_02_SAMLET +
              RA15_Q11_04_SAMLET + RA15_Q11_05_SAMLET

  # Structural path controlling for NA/PA
  T1_STRESS ~ QUANTDEM_lat + NA_lat + PA_lat
'

fit_sam_m1_quant <- sam(model = sam_m1_quant, data = data, sam.method = "local")
fit_sam_m2_quant <- sam(model = sam_m2_quant, data = data, sam.method = "local")

summary(fit_sam_m1_quant, standardized = TRUE)
summary(fit_sam_m2_quant, standardized = TRUE)


# --------------------------------------------------------------------------
# 2. Leadership Quality → T1 Job Satisfaction
# --------------------------------------------------------------------------

sam_m1_lead <- '
  # Measurement model
  NA_lat     =~ RA15_Q31_02 + RA15_Q31_06 + RA15_Q31_09 + RA15_Q31_10
```

```
  PA_lat     =~ RA15_Q31_01 + RA15_Q31_03 + RA15_Q31_15
  LEAD_lat   =~ RA15_Q21_05 + RA15_Q21_07 + RA15_Q21_11 + RA15_Q21_13

  # Zero-order structural path
  T1_JOBSAT ~ LEAD_lat
'

sam_m2_lead <- '
  # Measurement model
  NA_lat     =~ RA15_Q31_02 + RA15_Q31_06 + RA15_Q31_09 + RA15_Q31_10
  PA_lat     =~ RA15_Q31_01 + RA15_Q31_03 + RA15_Q31_15
  LEAD_lat   =~ RA15_Q21_05 + RA15_Q21_07 + RA15_Q21_11 + RA15_Q21_13

  # Structural path controlling for NA/PA
  T1_JOBSAT ~ LEAD_lat + NA_lat + PA_lat
'

fit_sam_m1_lead <- sam(model = sam_m1_lead, data = data, sam.method = "local")
fit_sam_m2_lead <- sam(model = sam_m2_lead, data = data, sam.method = "local")

summary(fit_sam_m1_lead, standardized = TRUE)
summary(fit_sam_m2_lead, standardized = TRUE)
```
