# Supplementary material for: Individual differences in affect: explaining work environment perceptions and later wellbeing
Source: Sci Rep. 2026 Jun 11;16:18178. doi: 10.1038/s41598-026-55924-9 (PMC13260830; doi:10.1038/s41598-026-55924-9)
Supplement: Supplementary file 2 — Supplementary Material 2 [file 41598_2026_55924_MOESM2_ESM.pdf]

```
#
==============================================================================
=======
# DATA PREPARATION SCRIPT
# "Individual Differences in Employees: How Affect Shapes Work Environment
#  Perceptions and Longitudinal Wellbeing"
#
==============================================================================
=======
# Source data: Danish Psychosocial Questionnaire (DPQ), two time points
# Pipeline:    Variable selection → Exclusion criteria → MICE imputation →
#              Reverse scoring → Composite scores → T1/T2 merge
#
==============================================================================
=======
# Note: Raw data are not publicly available (registry restrictions).
#       This script is provided for transparency and reproducibility of
#       analytical decisions.
#
==============================================================================
=======

library(tidyverse)
library(mice)
library(psych)
library(naniar)
library(haven)

# ── Load raw data
────────────────────────────────────────────────────────────

dpq_raw <- read_sas("data/raw_data.sas7bdat")
```

```
# ======================================================================
# TIME 1 — VARIABLE SELECTION
# ======================================================================

dpq_t1 <- dpq_raw %>%
  select(
    RA15_LOBENR,       # Participant ID
    RA15_UNDERSTYPE1,   # Survey type (used as non-imputed covariate)
    RA15_KOEN,         # Sex
    RA15_ALDERGRP,     # Age group
    RA15_Q01,          # Employment status
    RA15_Q02,          # Occupation type

    # Quantitative Demands (4 items)
    RA15_Q11_01_SAMLET, RA15_Q11_02_SAMLET,
    RA15_Q11_04_SAMLET, RA15_Q11_05_SAMLET,

    # Emotional Demands (4 items)
    RA15_Q11_11_SAMLET, RA15_Q11_12_SAMLET,
    RA15_Q11_13_SAMLET, RA15_Q11_14_SAMLET,

    # Role Clarity (4 items)
    RA15_Q10_06, RA15_Q10_07, RA15_Q10_09, RA15_Q10_10,
```

```
  # Employee Influence (4 items)
  RA15_Q09_10, RA15_Q09_11, RA15_Q09_12, RA15_Q09_13,

  # Teamwork / Cooperation (4 items)
  RA15_Q14_03, RA15_Q14_04, RA15_Q14_09, RA15_Q14_11,

  # Leadership Quality (4 items)
  RA15_Q21_05, RA15_Q21_07, RA15_Q21_11, RA15_Q21_13,

  # Perceived Stress (single item)
  RA15_Q33,

  # Negative Affect — PANAS items (4 items)
  RA15_Q31_02, RA15_Q31_06, RA15_Q31_09, RA15_Q31_10,

  # Positive Affect — PANAS items (3 items)
  RA15_Q31_01, RA15_Q31_03, RA15_Q31_15,

  # Job Satisfaction (single item)
  RA15_Q27,

  # Self-rated Health (single item)
  RA15_Q36
 )

#
=============================================================================
=======
# TIME 1 — EXCLUSION CRITERIA
```

```
#
======================================================================
=======

# Step 1: Remove extreme non-responders (>80% missing across T1 items)
# Rationale: Participants missing the majority of items cannot be meaningfully
#         imputed and likely did not engage with the survey.
dpq_t1 <- dpq_t1 %>%
  mutate(missing_pct = rowSums(is.na(.)) / ncol(.)) %>%
  filter(missing_pct < 0.80) %>%
  select(-missing_pct)

# Step 2: Employment status filter
# Retain codes 1–14 (employed/attached to workplace).
# Codes ≥15 indicate retirement, unemployment, or non-participation.
dpq_t1 <- dpq_t1 %>% filter(RA15_Q01 < 15)

# Step 3: Age group filter
# Exclude category 6 — identified as a data error with overlapping age ranges
# and a negligible number of cases.
dpq_t1 <- dpq_t1 %>% filter(RA15_ALDERGRP != 6)

#
======================================================================
=======
# TIME 1 — MULTIPLE IMPUTATION (MICE)
#
======================================================================
=======
# Overall missingness at T1 < 5%. MICE used to preserve statistical power
```

```
# and avoid bias from listwise deletion.

# Demographic/administrative variables excluded from the predictor matrix

# to prevent circularity.


imp_t1_init <- mice(dpq_t1, maxit = 0)

predM_t1    <- imp_t1_init$predictorMatrix


# Exclude non-substantive variables from imputation predictors

predM_t1[, c("RA15_LOBENR", "RA15_UNDERSTYPE1",

        "RA15_KOEN",   "RA15_ALDERGRP",

        "RA15_Q01",    "RA15_Q02")] <- 0


set.seed(1234)

imp_t1 <- mice(dpq_t1,

         maxit          = 5,

         predictorMatrix  = predM_t1,

         method         = imp_t1_init$method,

         print          = FALSE)

imp_t1 <- complete(imp_t1, action = 1)


#
=====================================================================
=======

# TIME 1 — REVERSE SCORING

#
=====================================================================
=======

# All work environment scales are scored so that higher = more favourable.

# Items originally scored in the unfavourable direction are reversed (6 - x).
```

PANAS items originally scored in the unfavourable direction reversed (7 - x).

```
# Work environment items (5-point scale: 1–5 → reversed to 5–1)
vars_reverse_t1 <- c(
  "RA15_Q11_01_SAMLET", "RA15_Q11_02_SAMLET",   # Quantitative Demands
  "RA15_Q11_04_SAMLET", "RA15_Q11_05_SAMLET",
  "RA15_Q11_11_SAMLET", "RA15_Q11_12_SAMLET",   # Emotional Demands
  "RA15_Q11_13_SAMLET", "RA15_Q11_14_SAMLET",
  "RA15_Q10_06",       "RA15_Q10_07",         # Role Clarity
  "RA15_Q10_09",       "RA15_Q10_10",
  "RA15_Q09_10",       "RA15_Q09_11",         # Employee Influence
  "RA15_Q09_12",       "RA15_Q09_13",
  "RA15_Q14_03",       "RA15_Q14_04",         # Teamwork
  "RA15_Q14_09",       "RA15_Q14_11",
  "RA15_Q21_05",       "RA15_Q21_07",         # Leadership Quality
  "RA15_Q21_11",       "RA15_Q21_13",
  "RA15_Q33"                               # Perceived Stress
)
imp_t1[, vars_reverse_t1] <- 6 - imp_t1[, vars_reverse_t1]

# PANAS items (6-point scale: 1–6 → reversed to 6–1)
vars_reverse_panas_t1 <- c(
  "RA15_Q31_01", "RA15_Q31_03",   # Positive Mood, Energetic
  "RA15_Q31_06", "RA15_Q31_09",   # Sad, Low Confidence
  "RA15_Q31_10"                # Guilt
)
imp_t1[, vars_reverse_panas_t1] <- 7 - imp_t1[, vars_reverse_panas_t1]
```

```
#
=============================================================================
# TIME 1 — COMPOSITE SCORES
#
=============================================================================

imp_t1 <- imp_t1 %>%
  mutate(
    RA15_QUANTITATIVEDEMANDS = rowSums(pick(RA15_Q11_01_SAMLET,
RA15_Q11_02_SAMLET,
                              RA15_Q11_04_SAMLET, RA15_Q11_05_SAMLET)),
    RA15_EMOTIONALDEMANDS    = rowSums(pick(RA15_Q11_11_SAMLET,
RA15_Q11_12_SAMLET,
                              RA15_Q11_13_SAMLET, RA15_Q11_14_SAMLET)),
    RA15_ROLECLARITY       = rowSums(pick(RA15_Q10_06, RA15_Q10_07,
                              RA15_Q10_09, RA15_Q10_10)),
    RA15_INFLUENCE         = rowSums(pick(RA15_Q09_10, RA15_Q09_11,
                              RA15_Q09_12, RA15_Q09_13)),
    RA15_COOPERATION       = rowSums(pick(RA15_Q14_03, RA15_Q14_04,
                              RA15_Q14_09, RA15_Q14_11)),
    RA15_LEADQUAL          = rowSums(pick(RA15_Q21_05, RA15_Q21_07,
                              RA15_Q21_11, RA15_Q21_13)),
    T1_NA                  = rowSums(pick(RA15_Q31_02, RA15_Q31_06,
                              RA15_Q31_09, RA15_Q31_10)),
    T1_PA                  = rowSums(pick(RA15_Q31_01, RA15_Q31_03, RA15_Q31_15))
  ) %>%
  rename(
    T1_STRESS = RA15_Q33,
```

```
    T1_JOBSAT = RA15_Q27,
    T1_HEALTH = RA15_Q36
  )

# 
======================================================================
=======
# TIME 2 — VARIABLE SELECTION
# 
======================================================================
=======

dpq_t2 <- dpq_raw %>%
  select(
    RA15_LOBENR,          # Participant ID (merge key)

    # Quantitative Demands T2 (4 items)
    RA15_Q11_01_SAMLET_FU, RA15_Q11_02_SAMLET_FU,
    RA15_Q11_04_SAMLET_FU, RA15_Q11_05_SAMLET_FU,

    # Emotional Demands T2 (4 items)
    RA15_Q11_11_SAMLET_FU, RA15_Q11_12_SAMLET_FU,
    RA15_Q11_13_SAMLET_FU, RA15_Q11_14_SAMLET_FU,

    # Role Clarity T2 (4 items)
    RA15_Q10_06_FU, RA15_Q10_07_FU, RA15_Q10_09_FU, RA15_Q10_10_FU,

    # Employee Influence T2 (4 items)
    RA15_Q09_10_FU, RA15_Q09_11_FU, RA15_Q09_12_FU, RA15_Q09_13_FU,
```

```
  # Teamwork / Cooperation T2 (4 items)
  RA15_Q14_03_FU, RA15_Q14_04_FU, RA15_Q14_09_FU, RA15_Q14_11_FU,

  # Leadership Quality T2 (4 items)
  RA15_Q21_05_FU, RA15_Q21_07_FU, RA15_Q21_11_FU, RA15_Q21_13_FU,

  # Perceived Stress T2 (single item)
  RA15_Q33_FU,

  # Negative Affect T2 — PANAS items (4 items)
  RA15_Q31_02_FU, RA15_Q31_06_FU, RA15_Q31_09_FU, RA15_Q31_10_FU,

  # Positive Affect T2 — PANAS items (3 items)
  RA15_Q31_01_FU, RA15_Q31_03_FU, RA15_Q31_15_FU,

  # Job Satisfaction T2 (single item)
  RA15_Q27_FU,

  # Self-rated Health T2 (single item)
  RA15_Q36_FU
 )

#
==============================================================================
# TIME 2 — EXCLUSION CRITERIA
```

```
#
==============================================================================
=======

# Remove extreme non-responders (>80% missing across T2 items)
dpq_t2 <- dpq_t2 %>%
  mutate(missing_pct = rowSums(is.na(.)) / ncol(.)) %>%
  filter(missing_pct < 0.80) %>%
  select(-missing_pct)

#
==============================================================================
=======
# TIME 2 — MULTIPLE IMPUTATION (MICE)
#
==============================================================================
=======

imp_t2_init <- mice(dpq_t2, maxit = 0)
predM_t2    <- imp_t2_init$predictorMatrix

# Exclude participant ID from imputation predictors
predM_t2[, "RA15_LOBENR"] <- 0

set.seed(1000)
imp_t2 <- mice(dpq_t2,
        maxit          = 5,
        predictorMatrix = predM_t2,
        method         = imp_t2_init$method,
        print          = FALSE)
```

```
imp_t2 <- complete(imp_t2, action = 1)


#
==========================================================================
=======
# TIME 2 — REVERSE SCORING
#
==========================================================================
=======


# Work environment items (5-point scale)
vars_reverse_t2 <- c(
  "RA15_Q11_01_SAMLET_FU", "RA15_Q11_02_SAMLET_FU",   # Quantitative Demands
  "RA15_Q11_04_SAMLET_FU", "RA15_Q11_05_SAMLET_FU",
  "RA15_Q11_11_SAMLET_FU", "RA15_Q11_12_SAMLET_FU",   # Emotional Demands
  "RA15_Q11_13_SAMLET_FU", "RA15_Q11_14_SAMLET_FU",
  "RA15_Q10_06_FU",       "RA15_Q10_07_FU",         # Role Clarity
  "RA15_Q10_09_FU",       "RA15_Q10_10_FU",
  "RA15_Q09_10_FU",       "RA15_Q09_11_FU",         # Employee Influence
  "RA15_Q09_12_FU",       "RA15_Q09_13_FU",
  "RA15_Q14_03_FU",       "RA15_Q14_04_FU",         # Teamwork
  "RA15_Q14_09_FU",       "RA15_Q14_11_FU",
  "RA15_Q21_05_FU",       "RA15_Q21_07_FU",         # Leadership Quality
  "RA15_Q21_11_FU",       "RA15_Q21_13_FU",
  "RA15_Q33_FU"                              # Perceived Stress
)
imp_t2[, vars_reverse_t2] <- 6 - imp_t2[, vars_reverse_t2]


# PANAS items (6-point scale)
```

```
vars_reverse_panas_t2 <- c(
  "RA15_Q31_01_FU", "RA15_Q31_03_FU",   # Positive Mood, Energetic
  "RA15_Q31_06_FU", "RA15_Q31_09_FU",   # Sad, Low Confidence
  "RA15_Q31_10_FU"                      # Guilt
)
imp_t2[, vars_reverse_panas_t2] <- 7 - imp_t2[, vars_reverse_panas_t2]

#
==========================================================================
=======
# TIME 2 — COMPOSITE SCORES
#
==========================================================================
=======

imp_t2 <- imp_t2 %>%
  mutate(
    T2_NA = rowSums(pick(RA15_Q31_02_FU, RA15_Q31_06_FU,
                RA15_Q31_09_FU, RA15_Q31_10_FU)),
    T2_PA = rowSums(pick(RA15_Q31_01_FU, RA15_Q31_03_FU, RA15_Q31_15_FU))
  ) %>%
  rename(
    T2_STRESS = RA15_Q33_FU,
    T2_JOBSAT = RA15_Q27_FU,
    T2_HEALTH = RA15_Q36_FU
  )

#
==========================================================================
=======
```

```
# MERGE T1 AND T2
# =============================================================================
# Left join retains all T1 participants; T2 variables are NA for non-completers.
# T2 participation is therefore not a strict inclusion criterion.

Completedata <- left_join(imp_t1, imp_t2, by = "RA15_LOBENR")

# =============================================================================
# SAVE FINAL DATASET
# =============================================================================
# write.csv(Completedata, "FINALDATA.csv", row.names = FALSE)
```
