# Supplementary material for: Individual differences in affect: explaining work environment perceptions and later wellbeing
Source: Sci Rep. 2026 Jun 11;16:18178. doi: 10.1038/s41598-026-55924-9 (PMC13260830; doi:10.1038/s41598-026-55924-9)
Supplement: Supplementary file 3 — Supplementary Material 3 [file 41598_2026_55924_MOESM3_ESM.docx]

# Appendix

**Table S1.** *Descriptive Statistics and Study Correlations between Work Environment (1-6) T1 and Employee Well-being (7-9) T2*

| Variables | *M* | *SD* | α | 1 | 2 | 3 | 4 | 5 | 6 | 7 | 8 | 9 | 10 |
| --- | --- | --- | --- | --- | --- | --- | --- | --- | --- | --- | --- | --- | --- |
| 1. Quantitative Demands T1 | 8.91 | 3.16 | .84 | — |  |  |  |  |  |  |  |  |  |
| 2. Emotional Demands T1 | 7.17 | 3.67 | .83 | .34 | — |  |  |  |  |  |  |  |  |
| 3. Job Role Clarity T1 | 12.33 | 2.68 | .82 | -.24 | -.11 | — |  |  |  |  |  |  |  |
| 4. Employee Influence T1 | 11.28 | 3.44 | .87 | .01 | -.07 | .35 | — |  |  |  |  |  |  |
| 5. Teamwork T1 | 11.52 | 2.85 | .82 | -.19 | -.09 | .42 | .33 | — |  |  |  |  |  |
| 6. Leadership T1 | 10.06 | 3.72 | .91 | -.20 | -.13 | .45 | .32 | .49 | — |  |  |  |  |
| 7. Job stress T2 | 2.47 | 1.02 | — | .36 | .25 | -.19 | -.13 | -.19 | -.17 | — |  |  |  |
| 8. Health T2 | 3.58 | 0.90 | — | -.11 | -.10 | .19 | .22 | .20 | .16 | -.34 | — |  |  |
| 9. Job Satisfaction T2 | 6.99 | 2.15 | — | -.18 | -.17 | .36 | .42 | .36 | .39 | -.35 | .30 | — |  |
| 10. Negative Affect T1 | 5.55 | 3.39 | .80 | .29 | .24 | -.34 | -.23 | -.31 | -.27 | .41 | -.30 | -.37 | — |
| 11. Positive Affect T1 | 12.00 | 2.44 | .77 | -.21 | -.17 | .35 | .28 | .34 | .32 | -.32 | .37 | .40 | -.73 |

*Note*. T1 *N* = 3,970, T2 follow-up 6 months later *N* = 2,375. *M* = Mean. *SD* = Standard deviation. α = Chronbach's alpha. Quantitative Demands, Emotional Demands, Job Role Clarity, Employee Influence, Teamwork, Leadership (scale 1-17); Job stress and Health (scale 1-5); Job Satisfaction (scale 0-10); Negative affect (scale 1-21); Positive affect (scale 1-16). After Bonferroni-correction *r* >= .10 was significant at *p* < .00027. In grey are work outcome variables 7, 8, 9, and 10.

**Table S2.** *Descriptive Statistics and Study Correlations between Employee Well-being T2 (1-3) and Negative Affect and Positive Affect T2*

| Variables | 1 | 2 | 3 | 4 |
| --- | --- | --- | --- | --- |
| 1. Job Stress T2 | — |  |  |  |
| 2. Health T2 | -.34 | — |  |  |
| 3. Job Satisfaction T2 | -.35 | .30 | — |  |
| 4. Negative Affect T2 | .61 | -.41 | -.51 | — |
| 5. Positive Affect T2 | -.51 | .49 | .57 | -.76 |

*Note*. T2 follow-up 6 months later *N* = 2,375. Job stress and Health (scale 1-5); Job Satisfaction (scale 0-10); Negative Affect (scale 1-21); Positive Affect (scale 1-16). All estimates were significant (*p* < .001). In grey are employee well-being variables 7, 8, and 9.

**Table S3***: Study Correlations between Negative Affect and Positive Affect Items T1 and T2*

| Items | 1 | 2 | 3 | 4 | 5 | 6 | 7 | 8 | 9 | 10 | 11 | 12 | 13 |
| --- | --- | --- | --- | --- | --- | --- | --- | --- | --- | --- | --- | --- | --- |
| 1. TenseT1 | — |  |  |  |  |  |  |  |  |  |  |  |  |
| 2. SadT1 | .42 | — |  |  |  |  |  |  |  |  |  |  |  |
| 3. LowconfidenceT1 | .42 | .62 | — |  |  |  |  |  |  |  |  |  |  |
| 4. GuiltT1 | .40 | .50 | .63 | — |  |  |  |  |  |  |  |  |  |
| 5. PositivemoodT1 | -.65 | -.56 | -.51 | -.38 | — |  |  |  |  |  |  |  |  |
| 6. EnergiticT1 | -.61 | -.44 | -.45 | -.37 | .67 | — |  |  |  |  |  |  |  |
| 7. TalkativeT1 | -.38 | -.50 | -.54 | -.44 | .48 | .42 | — |  |  |  |  |  |  |
| 8. TenseT2 | .48 | .28 | .31 | .30 | -.38 | -.38 | -.27 | — |  |  |  |  |  |
| 9. SadT2 | .36 | .43 | .42 | .33 | -.39 | -.33 | -.36 | .48 | — |  |  |  |  |
| 10. LowconfidenceT2 | .35 | .42 | .54 | .41 | -.38 | -.33 | -.40 | .45 | .65 | — |  |  |  |
| 11. GuiltT2 | .34 | .33 | .41 | .50 | -.30 | -.28 | -.30 | .45 | .50 | .59 | — |  |  |
| 12. PositivemoodT2 | -.41 | -.34 | -.34 | -.26 | .50 | .42 | .34 | -.69 | -.59 | -.50 | -.40 | — |  |
| 13. EnergeticT2 | -.41 | -.31 | -.35 | -.28 | .46 | .51 | .32 | -.65 | -.51 | -.47 | -.39 | .73 | — |
| 14. TalkativeT2 | -.33 | -.36 | -.38 | -.30 | .38 | .31 | .48 | -.44 | -.55 | -.54 | -.41 | .49 | .47 |

*Note.* Negative affect items: Tense, Sad, Low Confidence, and Guilt; Positive affect items: Positive mood, Energetic, and Talkative.

T1 N = 3,970 T2 follow-up 6 months later N = 2,375. T1 = Time baseline, T2 = 6-month follow-up.

**Table S4**: *Overview of Construct Composition*

| Measurements | Items |
| --- | --- |
| Quantitative Demands | 1. How often is it the case that you do not have time to complete all your work tasks? 2. How often do you receive unscheduled work tasks that place you under time pressure? 3. How often do you have deadlines that are hard to meet? 4. Do you get behind with your work? |
| Emotional Demands | 1. Are you placed in emotionally demanding situations at work? 2. As a result of your work, do you come into contact with people who oppose you or are aggressive towards you? 3. Do you have to deal with relationships at work that are emotionally challenging? 4. As a result of your work, do you have contact with people who are in difficult situations (e.g. people affected by a serious illness, accidents, grief, crises or social problems)? |
| Job Role Clarity | 1. Are there clear goals for your work tasks? 2. Do you know exactly what is expected of you at work? 3. Do you know when you have carried out your job well? 4. Do you know exactly what your responsibilities are? |
| Employee Influence | 1. Do you have any influence on how you carry out your work tasks? 2. Do you have sufficient authority to deal with the responsibilities you have in your work? 3. Is it possible for you to make important decisions about your work? 4. Do you have any influence on the order in which you carry out your work tasks? |
| Teamwork | 1. Do you and your colleagues help each other if someone has too much to do? 2. Is there a sense of community and cohesion between you and your colleagues? 3. Do you and your colleagues work well together when problems emerge that require cooperation among you? 4. Do you and your colleagues agree on what is most important in your work tasks? |
| Leadership | 1. Does your immediate supervisor give high priority to the well-being of employees in the workplace? 2. Is your immediate supervisor good at communicating clear goals for the work of you and your colleagues? 3. Is your immediate supervisor good at resolving conflicts? 4. Is your immediate supervisor good at motivating the employees? |
| Job Stress | 1. How often have you felt stressed within the last two weeks? |
| Health | 1. Overall, how do you perceive your health? |
| Job Satisfaction | 1. Overall, how satisfied are you with your job? |
| Negative Affect | 1. How much of the time in the last two weeks have you felt calm and relaxed? (Reverse) 2. How much of the time in the last two weeks have you been sad or upset? 3. How much of the time in the last two weeks have you had lower self-confidence? 4. How much of the time in the last two weeks have you had a bad conscience or feelings of guilt? |
| Positive Affect | 1. How much of the time in the last two weeks have you felt quiet or reserved? (Reverse) 2. How much of the time in the last two weeks have you been active and energetic? 3. How much of the time in the last two weeks have you been happy and in good mood? |
